# Report on Dental Literature

**Published:** 1872-12

**Authors:** W. H. Morgan


### Report on Dental Literature.

BY W. H. MORGAN.

*Read Before the Tennessee State Dental Association.*

The Chairman of your Committee on Dental Literature, not having had the assistance of any other member of the committee, would beg leave to make the following very brief and imperfect report, asking the leniency of his professional brethren.

There have been of recent date no new books added to our literature in this country, coming under the observation of you committee, except Wedl on the Pathology of the Teeth, translated from the German, with notes, by Prof. Hitchcock, of Boston. Not having opportunity to more than glance at its contents, we are not prepared to criticise its contents. The notes by Prof. Hitchcock are few and far between. Those who have read it pronounce it a valuable addition to our dental libraries, although the translating is badly done—the sense of the text being often marred. It is a book of some 300 pages, well-gotten up in library binding. There is a recent edition of Prof. Garretson's work, revised and enlarged, much better adapted to the wants of the dental student than his former editions.

Next is Bloxmus Laboratory Teaching, kindly spoken of by those competent to judge. Next comes "The Teeth and How to Save Them, by L. P. Meredith, M. D., D. D. S., of Cincinnati. This little work of 200 pages has received the compliment of being reprinted in London. One writer says of this book: "Although this work is offered particularly as a family dentist, giving the public all necessary information in regard to the teeth, yet dentists everywhere will find it to their interest to recommend and present, or sell it to their patients, for its tendency is to educate the public to decry quackery, to appreciate good work, to pay good prices, and to intelligently cooperate with the educated dentist." The *Cosmos* says: "We find much more to praise than to condemn, and wish a copy of it was in the hands of every family in the land." Your committee after glancing at this volume do not think very highly of it. A work issued in 1869, by Robert Arthur, M. D., D. D. S., of Baltimore, continues to attract a large share of attention. Its teachings are rather antiquated; its main doctrine being considered as exploded by a large majority of the most learned and advanced in the profession. Its style is vague often, and the cuts are very incorrect or untrue, if the author wishes to represent the teeth as we meet them in every day practice. On page eight, the writer says, by this system the pain usually attending dental operations may be entirely avoided. That all the teeth of every individual, with rare exceptions, may be saved. On page seventeen, he says of the enamel, the fibres are united by an intermediate substance, and the external surface is smooth and polished. The opposite of this is most often true. On page twenty-one he says: "As soon as the teeth make their appearance through the gums, they are subject to a process of decomposition, by which they are more or less rapidly and entirely destroyed, unless some means are employed to arrest its progress. Page twenty-three is so confused that he puts in a foot note saying he does not propose to be very accurate or minute, as he is writing for the general reader, the very person above all others he should be accurate for; and so on from beginning to end he is full of inaccuracies and errors. This being the case in his statement of facts, we are impressed with the idea that his observations are very unreliable. We would therefore insist that this mode of practice, or system, as he falsely calls it, should be very slowly adopted, and with great caution indeed. We do not believe as a whole it should be adopted at all, many of the ideas advanced, having long since been exploded, and abandoned by the best observers and practitioners in the profession. With all these and many more grave faults in this book, there is much in that portion devoted to hygiene, that is commendable, with one exception, the use of the file. With this single exception, this subject is treated in a most forcible manner, and its perusal must result in great good, both to the professional man and the general reader. We would especially commend his pains-taking manner of examining the teeth, with the view of removing the slightest decay, and of impressing upon the young patient the necessity of frequent examinations.

The character of our four leading journals has not materially changed within the last year. The *Cosmos* occupies a prominent place, having able original communications and judicious selections. It has lost nothing in interest under its "impersonal" editor. Next in order of value comes the DENTAL REGISTER, and while its editorials are short, and many of its original papers are of little value, yet it embodies much of the current literature of our profession, and no dentist can well afford to be without it. The Missouri *Journal* is edited with ability, contributing its share to progress in our profession. We regret to say the *American Journal of Dental Science,* the oldest of our journals, and the one which has contributed most to the dissemination of dental knowledge, has fallen behind in the struggle for usefulness, and although it has at its head an editor, both talented and capable, his editorials are few and far between, and do not often do much credit to their gifted author. And then its selections, or rather many of its original papers, for the last year or two are almost worthless, some of them quite so. Once in a while we have in its columns a valuable contribution to dental literature. In addition to the above we have one or two publications intended for gratuitous circulation. These are not of much value, nor are they exerting any considerable influence in or out of our ranks.

We have thus attempted to sketch hastily the character of our journals; and although they are not what they should be, and what we hope soon to see them, yet we think every dentist in the land, and especially every dentist in Tennessee, and most particulary and especially every dentist belonging to this association, should take all four of the above named periodicals. Other things being equal, the dentist who takes all of our journals and reads them carefully will be the superior of his neighbors who do not.

